# Chronic IL-1 Exposure Attenuates IL-1 Response and Alters Gene Expression Regulation While Maintaining Therapeutic Sensitivity in BCa Cell Lines

**DOI:** 10.3390/ijms27136039

**Published:** 2026-07-05

**Authors:** Rafah Falah, Roopal Dhar, Stephanie Yamauchi, Monica Bautista, Mohammed Kanchwala, Liu Yan, Dinesh Raju, Linyi Xu, Kylah Reliford, Afshan Nawas, Samrah Ali, Justin Fang, Ola Olaleye, Jyotsna Tera, Rana Abdelaziz, Reshmika Kanakala, Aniketh Sudunagunta, Subhash Eedarapali, Emmalee Burr, Basir S. Mansoor, Nicole Roos, Sydney Diep, Hiba Afaq, Niranjana Pillai Rajesh, Saanvi Manohar, Jennifer Odikpo, Abhinav K. Jain, Zhenyu Xuan, Chao Xing, Nikki A. Delk

**Affiliations:** 1 Department of Biological Sciences, The University of Texas at Dallas, Richardson, TX 75080, USA; rfalah@utdallas.edu (R.F.); roopal.dhar@utdallas.edu (R.D.); stephanieyamauchi@yahoo.com (S.Y.); mabautista1105@gmail.com (M.B.); linyi.xu@utdallas.edu (L.X.); kylah.reliford@utdallas.edu (K.R.); afshanfathima.nawas@utsouthwestern.edu (A.N.); samrah.ali18@gmail.com (S.A.); justin.fang02@gmail.com (J.F.); oolaleyems@gmail.com (O.O.); jyotsnatera@gmail.com (J.T.); rabdelaziz036@gmail.com (R.A.); reshmika.kanakala@gmail.com (R.K.); sudunaguntaaniketh@gmail.com (A.S.); subhash.arrowroot@gmail.com (S.E.); emmalee.l.burr@gmail.com (E.B.); basirmansoor12@gmail.com (B.S.M.); nicole.roos@emory.edu (N.R.); sydney_diep@yahoo.com (S.D.); hiba.afaq@utdallas.edu (H.A.); niranjanapillai.rajesh@utdallas.edu (N.P.R.); saanvi.manohar@utdallas.edu (S.M.); jenniferodikpo@gmail.com (J.O.); zhenyu.xuan@utdallas.edu (Z.X.); 2 Bioinformatics Lab, Eugene McDermott Center for Human Growth and Development, The University of Texas Southwestern Medical Center, Dallas, TX 75390, USA; mohammedmsk@gmail.com (M.K.); yan.liu.1@vumc.org (L.Y.); dinesh.ravindraraju@utsouthwestern.edu (D.R.); chao.xing@utsouthwestern.edu (C.X.); 3 Epigenetics & Molecular Carcinogenesis, Center for Cancer Epigenetics, The University of Texas MD Anderson Cancer Center, Houston, TX 77030, USA; ajain@mdanderson.org

**Keywords:** breast cancer, estrogen receptor, progesterone receptor, Interleukin-1, acute inflammation, chronic inflammation

## Abstract

Chronic inflammation is a hallmark of the breast cancer tumor microenvironment and is also known to be associated with disease progression and therapeutic response. Interleukin-1 (IL-1) signaling has been widely studied in breast cancer biology; however, the long-term effect of sustained IL-1 exposure on hormone receptor-positive breast cancer cells remain poorly understood. In this study, we investigated how chronic IL-1 exposure influences inflammatory response, hormone dependency, and therapeutic sensitivity in ERα+/PR+ breast cancer models, MCF7 and T47D. Chronic IL-1 exposure attenuated response to subsequent acute IL-1 treatment, but the chronically exposed cells remained sensitive to serum deprivation, retained dependence on estrogen or progesterone receptor signaling, and responded robustly to endocrine and chemotherapeutic treatments. Extensive changes in basal gene expression and histone modification revealed that chronic IL-1 exposure alters transcriptional reprogramming and chromatin remodeling. Together, these findings demonstrate that chronic IL-1 signaling drives selective inflammatory response in hormone receptor-positive MCF7 and T47D breast cancer cells. This work underscores the continued therapeutic relevance of hormone receptor-targeted strategies in chronically inflamed tumors and provides insight into how sustained inflammatory stress shapes tumor behavior and gene regulation predicted to promote tumor progression.

## 1. Introduction

Breast cancer (BCa) is a leading cause of cancer-related death among women worldwide [1]. Due to the intricate pathogenesis of the disease, it poses major obstacles in its treatment advancement and prevention [2]. Estrogen receptor α (ERα) and progesterone receptor (PR) are nuclear hormone receptors that drive the growth and progression of BCa [3,4]. Around two-thirds of patient tumors are hormone-receptor-positive (i.e., ERα+/PR+), and are therefore treated with endocrine therapies including selective estrogen receptor modulators (e.g., tamoxifen) and selective estrogen-receptor degraders (e.g., fulvestrant) that block ERα and PR activity [5]. Unfortunately, endocrine therapies are initially effective, but many tumors eventually recur or progress, highlighting the need to better understand external signals from the tumor microenvironment (TME) that shape how cancer cells adapt and respond to therapy.

The TME is replete with inflammatory cytokines that regulate tumorigenesis and tumor progression. Interleukin-1 (IL-1) is a widely studied inflammatory cytokine that exists in two biologically active forms, IL-1α and IL-1β, both of which bind to the same receptor, IL-1 receptor type 1 (IL-1R1), leading to recruitment of the co-receptor, Interleukin-1 Receptor Accessory Protein (IL-1RAcP), to activate the NF-κB pathway [6,7]. Although both IL-1 isoforms bind to the same receptor and show similar biological functions, they also have distinct functions, are encoded by distinct genes and differ in their regulation [8,9,10]. IL-1α is produced constitutively by multiple cell types and is secreted as an alarmin during necrosis. IL-1α also functions as a dual cytokine, localizing to the nucleus to regulate gene expression. IL-1β is secreted primarily by immune cells during inflammatory responses and requires cleavage by caspase-1 for activation and secretion. Both IL-1α and IL-1β are functionally and clinically relevant in cancer, including in BCa [10,11,12,13]. Both IL-1 isoforms promote EMT, stemness, senescence, angiogenesis, and metastasis and correlate with advanced BCa stage, basal subtype, and metastasis.

Acute inflammation is primarily anti-tumorigenic [14], and in kind, acute exposure to IL-1 inhibits the proliferation of ERα+/PR+ breast cancer cells [15]. However, prolonged exposure to inflammatory cytokines can lead to selective pressure that supports tumor growth and progression [14]. Using the human prostate cancer (PCa) cell line, LNCaP, we previously discovered that chronic exposure to IL-1 selects for cells that evolve insensitivity to IL-1 signaling and acquire androgen receptor (AR) independence and resistance to AR-targeted therapy [16]. Thus, IL-1 may contribute to PCa progression and hormone therapy resistance in patients. Because BCa and PCa are both hormone-receptor-driven cancers [17,18], chronic IL-1 exposure may likewise select for BCa cells that evolve IL-1 insensitivity and acquire endocrine resistance. In this study, we generated novel chronic IL-1 subline models using MCF7 and T47D human BCa cell lines to test IL-1 sensitivity, hormone receptor dependence, and chemotherapy sensitivity, and to identify changes in gene expression and epigenetic marks. Together, our studies aim to define how sustained inflammatory signaling influences tumor cell adaptation, including how chronic inflammation can lead to epigenetic modifications that underly gene regulation.

## 2. Results

### 2.1. Chronic IL-1 Selects for BCa Cells That Evolve Attenuated Response to Acute IL-1 Exposure

**Figure 1:**
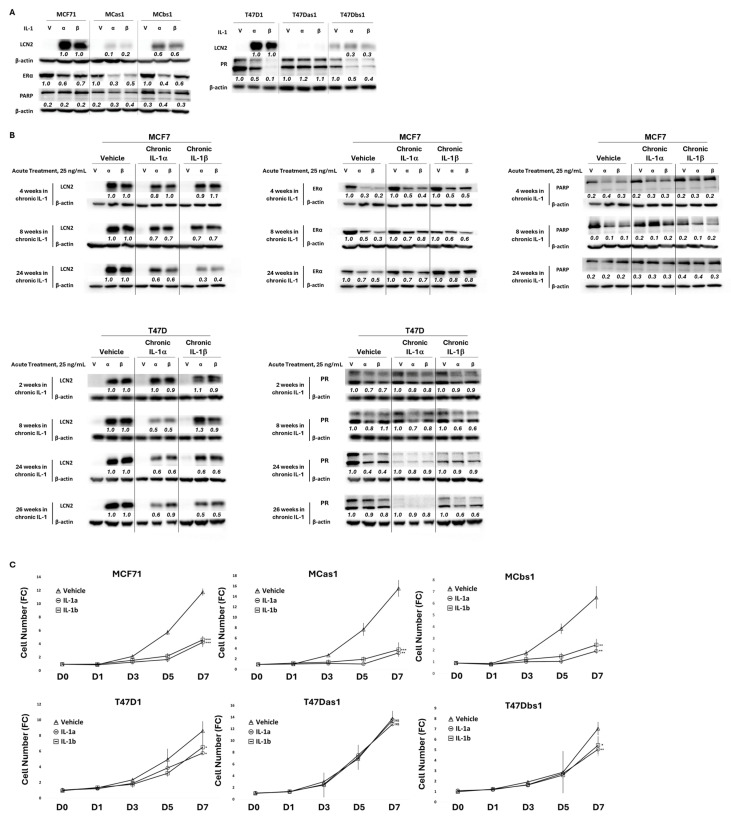
Chronic IL-1 selects for BCa cells that both lose and retain acute IL-1 responses. (**A**) MCF71 and T47D1 parental control cell lines, and their respective corresponding chronic IL-1 sublines, MCas1, MCbs1, T47Das1, and T47Dbs1, were treated acutely for 4 days with 25 ng/mL of IL-1α or IL-1β. Protein was isolated and Western blot was performed for ERα and PR hormone receptors and the canonical IL-1-induced gene, *Lipocalin 2* (*LCN2*), as a surrogate for IL-1 sensitivity. In comparison to parental control cells, the MCF7 and T47D chronic IL-1 sublines show attenuated response to acute IL-1 repression of LCN2 but, with the exception of T47Das1, maintain sensitivity to acute IL-1 repression of ERα and PR. MCF7 parental and subline cells were also analyzed for PARP cleavage, an indicator of apoptosis. Acute IL-1 does not induce considerable PARP cleavage, suggesting the acute IL-1 is not appreciably cytotoxic to MCF7 parental or subline cells. (**B**) MCF7 or T47D cell lines were cultured in 0.5 ng/mL of IL-1α or IL-1β chronically for 2, 4, 8, 24, and/or 26 weeks, and at each timepoint, cells were collected and replated in normal growth medium for 3 days to allow cells to attach in culture. Once attached in culture, cells were treated acutely for 4 days with 25 ng/mL of IL-1α or IL-1β, and protein was isolated to assess LCN2, ERα or PR accumulation, or PARP cleavage. Western blot analysis shows that within 8 weeks of chronic IL-1 exposure, cells begin to evolve an attenuated response to acute IL-1 treatment. (**C**) MCF71 and T47D1 parental and subline cells were treated with vehicle control, 25 ng/mL of IL-1α, or 25 ng/mL of IL-1β for 7 days. Attached cells were fixed and their nuclei were stained with the DAPI. DAPI-stained cells were counted before IL-1 treatment (day 0, “D0”) and at 1, 3, 5, and 7 days after treatment, and the average fold change (FC) in cell counts was plotted. IL-1-treated MCF71, MCas1, and MCbs1 cells show reduced proliferation rate, while IL-1 had comparably little or no effect on T47D1 parental and subline cells. Cell counts were normalized to day 0 for each cell line. *n* = 3 biological replicates; error bars = ±STDEV; *p*-value = * ≤0.05, ** ≤0.005, *** ≤0.0005. NS = not significant. Western blot densitometry was performed using ImageJ (v 1.53k) and protein bands normalized to β-actin loading control. LCN2 bands were further normalized to the parental control for each cytokine, ERα and PR bands were further normalized to the vehicle control within the cell line or subline, and the ratio of cleaved/full-length PARP was determined.

We previously reported that chronic IL-1 exposure selects for cells that evolve IL-1 insensitivity in androgen receptor (AR)-positive LNCaP, C4-2, and MDA-PCa-2b human prostate cancer (PCa) cell lines [16,19]. To determine if chronic IL-1 exposure has the same effect on hormone-receptor-positive breast cancer cells, we exposed MCF7 and T47D cells to IL-1 chronically for several months to establish chronic IL-1 sublines (described in “Materials and Methods”) and then assayed subline sensitivity to acute IL-1 treatment. To test for sensitivity to acute IL-1, parental control cell lines, referred to as MCF71 and T47D1, and their respective corresponding chronic IL-1 sublines, MCas1, MCbs1, T47Das1, and T47Dbs1, were treated acutely for 4 days with 25 ng/mL of IL-1α or IL-1β. Using the canonical IL-1-induced gene, *Lipocalin 2* (*LCN2*), as a surrogate for IL-1 sensitivity, we found that, in comparison to parental control cells, the MCF7 and T47D chronic IL-1 sublines show attenuated response to acute IL-1 treatment (Figure 1A). The attenuation mechanism requires thorough investigation, but RNA sequencing shows that among the sublines, MCas1, MCbs1, T47Das1, and T47Dbs1, *IL-1R1* or *IL-1RAcP* mRNA levels are not significantly differentially expressed (Table S1, Tab B and D), suggesting that the attenuated IL-1 response is not due to downregulation of the IL-1 receptor complex. For rigor, we generated a second set of chronic IL-1 sublines and found, similarly, that the chronic IL-1 sublines show attenuated *LCN2* induction in acute IL-1 treatment (Figure S1A).

To better understand the timing of when chronically exposed cells develop reduced sensitivity to acute treatment, we treated MCF7 or T47D cells chronically with 0.5 ng/mL of IL-1α or IL-1β for 2, 4, 8, 24, and/or 26 weeks, and at each timepoint, we assessed LCN2 accumulation in response to a 4-day acute, 25 ng/mL IL-1α or IL-1β treatment. Attenuated cell response to acute IL-1 treatment is detectable within 8 weeks of chronic IL-1 exposure in MCF7 and T47D cells (Figure 1B). Together, our results reveal that attenuated sensitivity to acute IL-1 is a conserved, reproducible response to chronic IL-1 exposure.

### 2.2. Acute IL-1 Repression of Hormone Receptors and Cell Proliferation Is Retained in the BCa Chronic IL-1 Sublines

**Figure 2:**
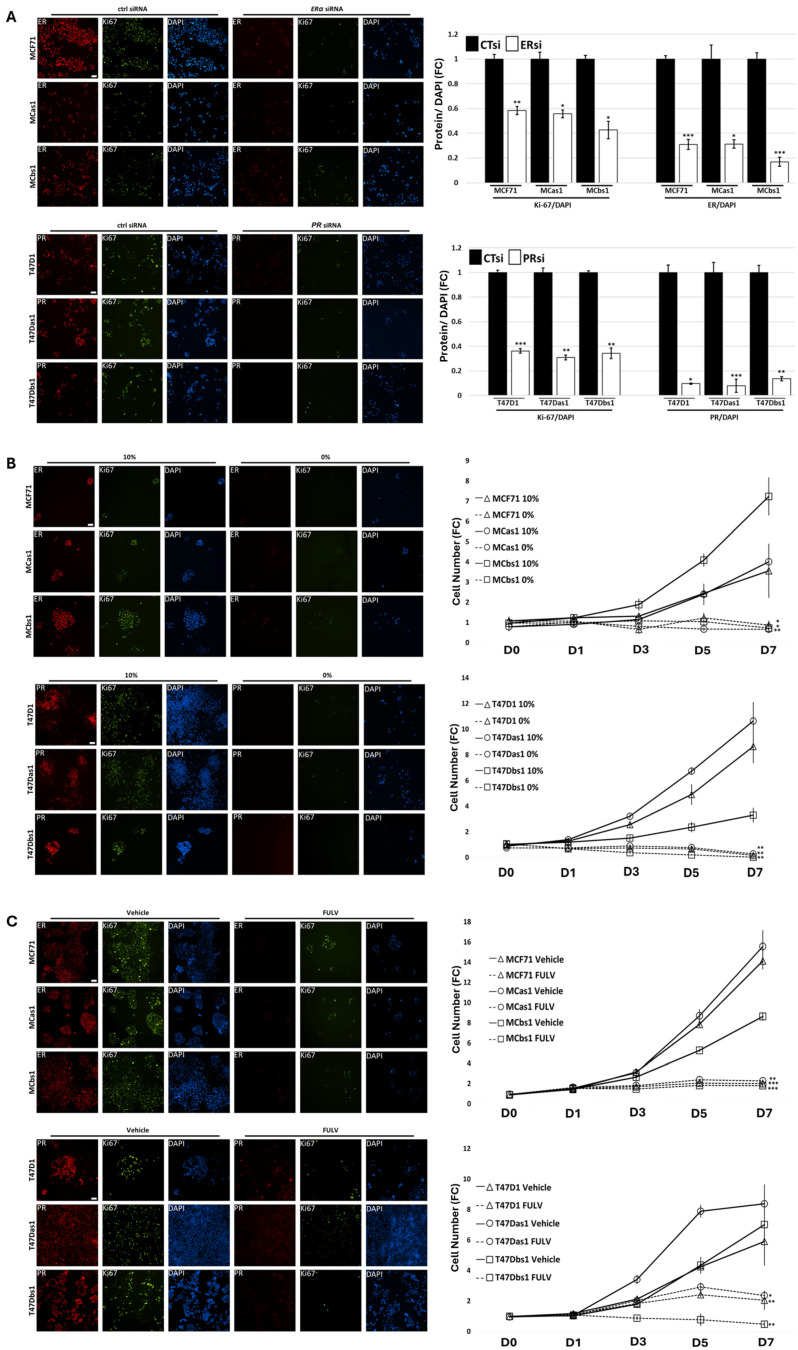
Chronic IL-1 sublines maintain dependency on ERα and PR hormone receptors. MCF71, T47D1, and subline cells were (**A**) transfected with 70 nM control siRNA, *ERα* siRNA, or *PR* siRNA for 4 days; (**B**) grown in 10% or 0% serum for 0, 1, 3, 5, and 7 days; or (**C**) treated with 100 nM fulvestrant (FULV) for 0, 1, 3, 5, and 7 days. Treated cells were fixed, DAPI-stained, and co-immunostained for ERα or PR and the proliferation maker, Ki67. To determine the treatment effect on cell proliferation, the ratio cells positive for ERα/DAPI, PR/DAPI, or Ki67/DAPI was plotted (**A**) or the number of DAPI-stained cells was counted over time (**B**,**C**). siRNA, serum starvation, and FULV treatment reduce ERα, PR, and Ki67 nuclear accumulation and proliferation in MCF71, T47D1, and subline cells. DAPI ratios were normalized to control siRNA and cell counts were normalized to day 0 for each cell line. *n* = 3 biological replicates; error bars = ±STDEV; *p*-value = * ≤0.05, ** ≤0.005, *** ≤0.0005. Images: scale bar = 100 μm.

Acute IL-1 represses AR, estrogen receptor alpha (ERα), and progesterone receptor (PR) mRNA and protein accumulation in BCa and PCa human cell lines [5,19,20,21,22]. However, we discovered that under chronic IL-1 exposure, PCa cell lines eventually restore AR accumulation and subsequently evolve resistance to acute IL-1-induced *AR* repression [16,19]. Likewise, under chronic IL-1 exposure, MCF7 and T47D cells restore ERα and PR accumulation, but contrary to what we observed for LNCaP and MDA-PCA-2b PCa cell line backgrounds, the MCF7 and T47D chronic IL-1 sublines can maintain sensitivity to acute IL-1 repression of ERα and PR accumulation (Figure 1A,B and Figure S1A). Interestingly, we have repeatedly observed that PR levels become increasingly repressed during chronic exposure specific to T47D cells treated with IL-1α (Figure 1B), but PR re-emerge following removal from chronic IL-1α exposure (Figure 1A, Figure 2 and Figures S1A and S2A). This result reveals that, specific to the cell line background, chronic IL-1 exposure can differentially impact expression of different IL-1 target genes, and IL-1α and IL-1β can also have differential effects on shared target genes.

Acute IL-1 treatment is cytotoxic and cytostatic for PCa cell lines [16,19,23], while chronic IL-1-exposed PCa cells evolve insensitivity to acute IL-1-induced cytotoxicity and cytostasis [16,19,24]. We found that treating cells acutely for 1, 3, 5, and 7 days with 25 ng/mL of IL-1α or IL-1β reduces the proliferation rate of MCF7 parental cells but is not considerably cytotoxic (Figure 1A–C and Figure S1A,B). Thus, unlike the PCa chronic IL-1 sublines [16], the MCF7 chronic IL-1 sublines maintain sensitivity to acute IL-1-induced cytostasis (Figure 1C and Figure S1B). Finally, T47D parental and T47D chronic IL-1 sublines treated acutely with 25 ng/mL of IL-1α or IL-1β show only modest or no reduction in proliferation rate (Figure 1C and Figure S1B). Taken together, acute IL-1 can be cytotoxic and/or cytostatic, but maintaining acute sensitivity following chronic IL-1 exposure is dependent on the cell line background.

### 2.3. BCa Chronic IL-1 Sublines Maintain Dependency on ERα and PR Hormone Receptors

We [16] and others [21] have shown that chronic IL-1 exposure can induce AR independence in the LNCaP PCa cell line. To determine if chronic IL-1 exposure can also confer ERα or PR independence in MCF7 and T47D cells, we subjected parental and chronic IL-1 subline cells to hormone-receptor-targeting treatments that block cell proliferation—hormone receptor silencing [25], serum starvation [26,27], or the anti-estrogen, fulvestrant [28]. *ERα* or *PR* siRNA for 4 days (Figure 2A and Figure S2A); serum starvation for 1, 3, 5, and 7 days (Figure 2B and Figure S2B); or fulvestrant treatment for 1, 3, 5, and 7 days (Figure 2C and Figure S2B) each reduce ERα or PR nuclear accumulation in parental and subline cells, concomitant with a reduction in cell proliferation. These data indicate that, unlike the LNCaP chronic IL-1 sublines [16,29], the MCF7 and T47D chronic IL-1 sublines maintain hormone receptor dependency for cell growth.

### 2.4. BCa Chronic IL-1 Sublines Remain Sensitive to Chemotherapy

**Figure 3:**
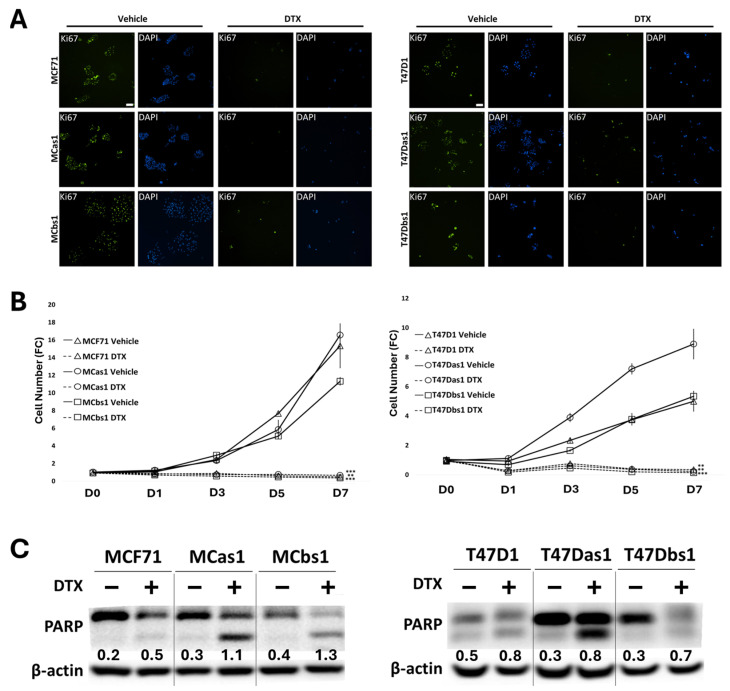
BCa chronic IL-1 sublines remain sensitive to chemotherapy. (**A**) MCF71, T47D1, and subline cells were treated with 10 nM docetaxel (DTX) for 3 days and co-stained with Ki67 and DAPI to determine the effect on proliferation. (**B**) MCF71, T47D1, and subline cells were treated with 10 nM DTX for 0, 1, 3, 5, and 7 days. Fixed, DAPI-stained cells were counted before treatment (day 0, “D0”) and at 1, 3, 5, and 7 days after treatment, and the average fold change (FC) in cell counts was plotted. (**C**) MCF71, T47D1, and subline cells were treated for 3 days with 10 nM DTX and analyzed for PARP cleavage by Western blot to assay for apoptosis. DTX treatment reduces Ki67 staining and DAPI cell counts over time, and induces PARP cleavage in the MCF71, T47D1, and sublines. Cell counts were normalized to day 0 for each cell line. *n* = 3 biological replicates; error bars = ±STDEV; *p*-value = ** ≤0.005, *** ≤0.0005. Images: scale bar = 100 μm. Western blot densitometry was performed using Image J and protein bands normalized to β-actin loading control, and the ratio of cleaved/full length PARP was determined.

Acute IL-1 exposure (i.e., 24–96 h) has been shown to induce resistance to the chemotherapeutic, docetaxel, in lung and head and neck cancer models [30,31]. Docetaxel, at 10 nM concentration, is sufficient to begin inducing MCF7 [32] and T47D [33] apoptosis in vitro after 24–48 h. Therefore, to test if MCF7 and T47D chronic IL-1 sublines acquire docetaxel resistance, parental and subline cells were treated with 10 nM docetaxel for 1, 3, 5, and 7 days and were assessed for cell death and proliferation. Docetaxel inhibited cell viability and proliferation in parental and chronic IL-1 subline cells (Figure 3 and Figure S3). Thus, chronic IL-1 exposure does not confer docetaxel resistance in MCF7 and T47D cells.

### 2.5. Chronic IL-1 Expsoure Causes Changes in Basal Gene Expression in MCF7 and T47D BCa Cells

Chronic inflammatory response differs mechanistically from acute response [14]. Therefore, we wanted to identify which genes were altered by acute versus chronic IL-1 exposure. We performed RNA sequencing (RNA-seq) and differential gene expression analysis for the parental (MCF71, T47D1) cells treated acutely with 25 ng/mL of IL-1α or 25 ng/mL of IL-1β for 4 days and for the chronic IL-1 sublines (MCas1, MCbs1, T47Das1, T47Dbs1) grown in vehicle control only. At the time of sequencing, the sublines had been out of chronic IL-1 treatment and moved to and cultured in normal growth media for at least 20 weeks (described in “Materials and Methods”) in order to establish stable sublines and to assess stable differentially expressed genes (DEG), rather than transient responses. Finally, Over-Representation Analysis (ORA, *p*-value ≤ 0.05) gene ontology and Gene Set Enrichment Analysis (GSEA, top 15 enriched categories, FDR ≤ 0.25) [34] gene ontology were performed for DEGs to predict regulated biological processes and signaling pathways.

RNA sequencing reveals that MCF7 and T47D cell lines show largely distinct gene expression patterns in response to IL-1 treatment that are predicted to converge on similar biological processes (Table S1). Acute IL-1 targets 1530 genes in MCF7 cells and 238 genes in T47D cells, with 96 target genes shared by MCF7 and T47D (Table S1, Tab A, C, E). Despite largely distinct expression patterns, ORA gene ontology predicts that acute IL-1 regulates inflammatory and immune responses in both MCF7 and T47D cell lines (Table S1, Tab F, G, H). Likewise, chronic IL-1 exposure leads to distinct gene expression patterns in MCF7 and T47D cells (Table S1). Chronic IL-1 exposure alters basal expression of 1417 genes in MCF7 cells and 2113 genes in T47D cells, with 289 of the altered genes shared by MCF7 and T47D (Table S1, Tab B, D, E). Despite largely distinct expression patterns, ORA gene ontology predicts that chronic IL-1 exposure alters cell cycle regulation in both MCF7 and T47D cell lines (Table S1, Tab I, J, K). Finally, acute and chronic IL-1 exposure led to distinct expression patterns in MCF7 and T47D cell lines. Of the 1530 and 1417 genes altered in MCF7 cells by acute and chronic IL-1 exposure, respectively, 405 genes are shared and predicted to regulate multiple processes including biomolecule transport (Table S1, Tab E and L), 1125 genes are unique to acute IL-1 treatment and predicted to regulate inflammatory and immune response (Table S1, Tab E and M), and 1012 genes are unique to chronic IL-1 treatment and predicted to regulate the cell cycle (Table S1, Tab E and N). Of the 238 and 2113 genes altered in T47D cells by acute and chronic IL-1 exposure, respectively, 103 genes are shared and predicted to regulate inflammatory and immune response (Table S1, Tab E and O), 135 genes are unique to acute IL-1 treatment and predicted to regulate cytokine signaling and immune response (Table S1, Tab E and P), and 2010 genes are unique to chronic IL-1 treatment and predicted to regulate the cell cycle (Table S1, Tab E and Q). In support of ORA GO, GSEA GO shows that acute IL-1α and IL-1β significantly enrich for upregulated genes that control NF-kB and inflammatory signaling in both MCF7 and T47D cell lines and also significantly enrich for downregulated genes that control estrogen response and DNA replication in MCF7 cells and estrogen response and organ development in T47D cells (Table S1, Tab V–CC). As also determined with ORA above, GSEA predicts chronic IL-1α and/or IL-1β exposure to enrich for upregulated genes that regulate the DNA replication and cell cycle in MCF7 and T47D cells and to also enrich for downregulated genes that regulate steroid hormone metabolism in MCF7 cells and organ development and secretion in T47D cells (Table S1, Tab DD–KK). Together, these data reveal that acute versus chronic IL-1 signaling drives genetic reprogramming that is context-specific (e.g., cell line background) and associated with divergent (e.g., inflammation versus cell cycle) downstream gene regulatory pathways. In addition, despite cell-line-specific genetic reprogramming, the predicted response elicited by acute IL-1 or by chronic IL-1 treatment is conserved across cell line backgrounds.

**Figure 4:**
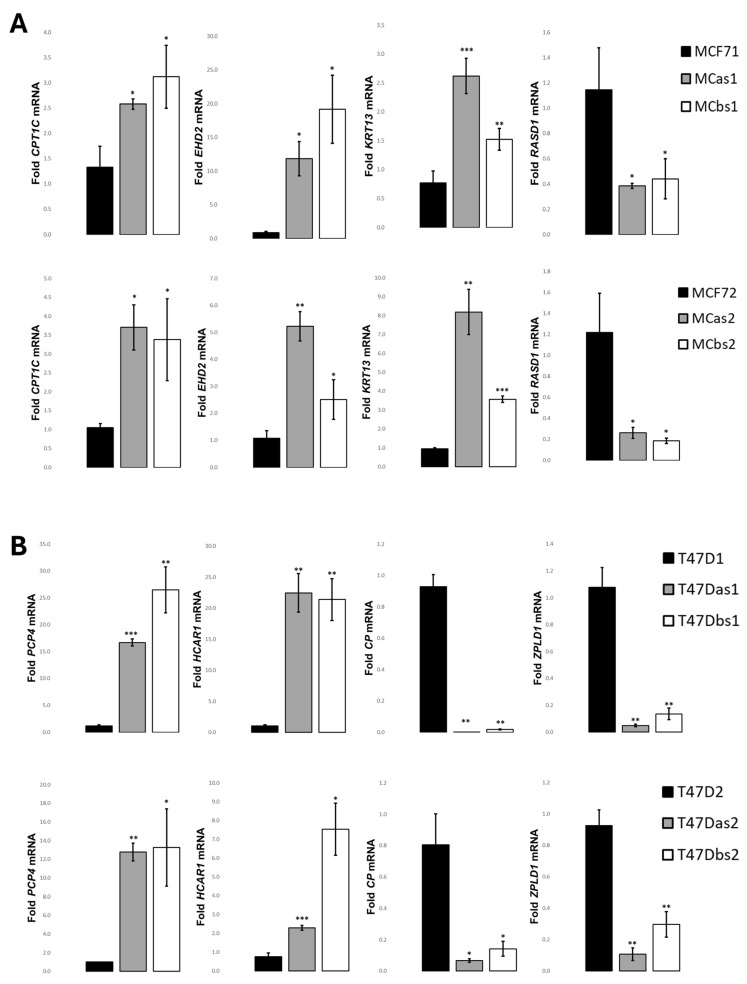
RT-qPCR confirmation of basally high and basally low expression for select genes identified by RNA-sequencing. (**A**) MCF71, MCas1, and MCbs1, and MCF72, MCas2, and MCbs2 were analyzed for the basal mRNA levels of the basally upregulated (*CPT1C*, *EHD2*, and *KRT13*) and basally downregulated (*RASD1*) genes by RT-qPCR. (**B**) T47D1, T47Das1, and T47Dbs1, and T47D2, T47Das2, and T47Dbs2 cells were analyzed for basal mRNA levels of the basally upregulated (*PCP4* and *HCAR1)* and basally downregulated (CP and ZPLD1) genes by RT-qPCR. Gene expression patterns for the select genes are conserved in both sets (1 and 2) of chronic IL-1 sublines. At the time of qPCR analysis, following chronic IL-1 exposure, MCas1, MCas2, MCbs1, and MCbs2 were out of IL-1 for 28 weeks; T47Das1 and T47Dbs1 were out of IL-1 for 52 weeks and 20 weeks, respectively; and T47Das2 and T47Dbs2 were out of IL-1 for 24 weeks. *n* = 3 biological replicates; error bars = ±STDEV; *p*-value = * ≤0.05, ** ≤0.005, *** ≤0.0005. Fold mRNA levels are normalized to parental control (MCF71, MCF72, T47D1, T47D2).

To begin to assess if chronic IL-1 gene regulation is consistent across both independently generated subline sets, we selected representative genes implicated in cancer progression and validated their basal expression by RT-qPCR. For MCF71 sublines, we chose the basally high genes, *CPT1C*, *EHD2* and *KRT13*, which are involved in tumorigenesis and metastasis [35,36,37], and the basally low gene, *RASD1*, which is involved in apoptosis regulation [38], and confirmed consistent gene expression patterns in both sets of sublines (MCas1, MCbs1, MCas2, and MCbs2) (Figure 4A). For T47D1 sublines, we selected the basally high genes, *PCP4* and *HCAR1*, which are involved in metastasis [39,40], and the basally low genes, *CP*, which is implicated in ferroptosis [41], and *ZPLD1*, which has been linked with poorer prognosis in cancer patients [42]. Both sets of sublines (T47Das1, T47Dbs1, T47Das2, and T47Dbs2) demonstrated consistent expression patterns (Figure 4B). Thus, chronic IL-1 appears to drive reproducible changes in gene expression.

### 2.6. Chronic IL-1 Expsoure Alters Histone Modifications in MCF7 BCa Cells

**Figure 5:**
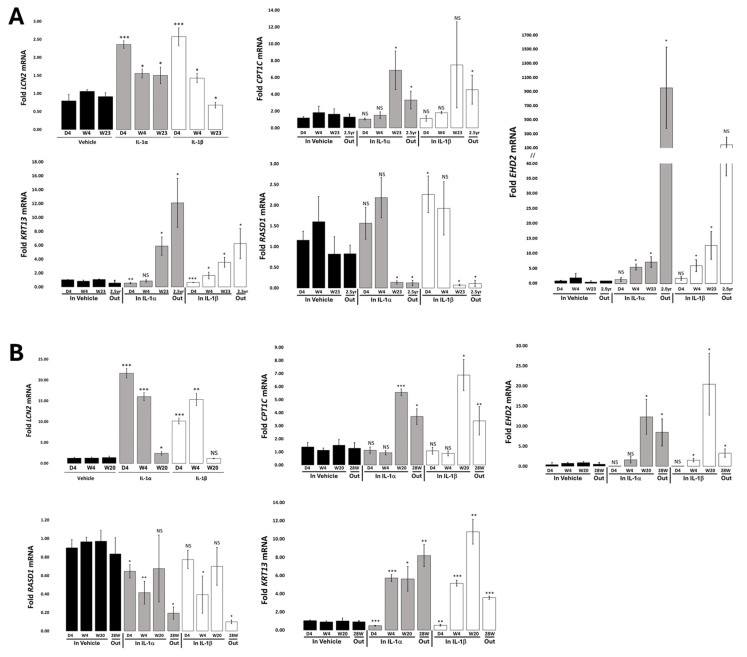
mRNA levels of select genes show conserved, predictable time-dependent basal expression patterns in chronic IL-1-exposed MCF7 cells. RT-qPCR was used to determine *LCN2*, *CPT1C*, *EHD2*, *KRT13*, and *RASD1* mRNA levels for (**A**) MCF71 parental cells grown in vehicle control, 0.5 ng/mL of IL-1α, or 0.5 ng/mL of IL-1β for 4 days, 4 weeks, or 23 weeks, or after 2.5 years of culturing in normal growth medium following removal from IL-1, or (**B**) MCF72 parental cells grown in vehicle control, 0.5 ng/mL of IL-1α, or 0.5 ng/mL of IL-1β for 4 days, 4 weeks, or 20 weeks, or after 28 weeks of culturing in normal growth medium following removal from IL-1. *n* = 3 biological replicates; error bars = ±STDEV; *p*-value = * ≤0.05, ** ≤0.005, *** ≤0.0005. NS = not significant. Attenuated IL-1 sensitivity and changes in basal gene expression can be detected as early as 4 weeks and by 20 or 23 weeks in IL-1 and maintain relative basal expression patterns after long-term culturing in normal growth medium. Fold mRNA levels are normalized to day 4 within each treatment.

RNA-seq reveals that cell lines chronically exposed to IL-1 acquire changes in basal gene expression (Table S1, Tab B and D) [16,19]. To determine the timing at which chronically exposed cells begin to show changes in basal gene expression, we analyzed select genes in MCF7 cells exposed to IL-1 acutely for 4 days and chronically for 4, 20 or 23 weeks. For rigor, we performed the time course experiment twice (MCF71, MCF72). Using *LCN2* upregulation as a surrogate for IL-1 signaling, we found that attenuated IL-1 response can be detected as early as 4 weeks and by 20 or 23 weeks of chronic IL-1 exposure along with basally high levels of *CPT1C*, *EHD2*, and *KRT13* and basally low levels of *RASD1* (Figure 5); after 2.5 years of culturing MCF71 chronic IL-1 subline cells in normal growth medium or 28 weeks of culturing MCF72 chronic IL-1 sublines in normal growth medium, *CPT1C*, *EHD2*, and *KRT13* remain basally high and *RASD1* remains basally low (Figure 5). Thus, chronic IL-1 signaling can drive predictable, conserved, stable genetic reprogramming.

Given the predictable, conserved, stabile effect of chronic IL-1 on gene expression, we reasoned that chronic IL-1 exposure alters chromatin state. Thus, to begin to understand how chronic IL-1 exposure regulates gene expression we identified changes in enrichment of histone modifications (histone marks) in the MCF71 chronic IL-1 sublines that had been cultured and passaged over the course of 2.5 years in normal growth medium (described in “Materials and Methods”). We used chromatin immunoprecipitation sequencing (ChIP-seq) to assess differential binding for H3K4me3 (active promoters), H3K4me1 (active enhancers), H3K27ac (active promoters and enhancers), and H3K27me3 (silenced genes) [43,44] (log2 FC ≥ 1.0 or ≤−1.0, and *p*-value ≤ 0.05) in MCas1 and MCbs1 chronic IL-1 subline cells versus MCF71 parental cells. Each histone mark analyzed shows differential binding in MCas1 and MCbs1 versus MCF71 parental control in promoters, UTRs, exons, introns, and distal intergenic regions, and the modifications are annotated for both shared and distinct sets of genes between the sublines (Table S2, Tab A–P). Taken together, chronic IL-1α and IL-1β exposure alters genome-wide histone modifications that are associated with chromatin remodeling and gene expression regulation.

**Figure 6:**
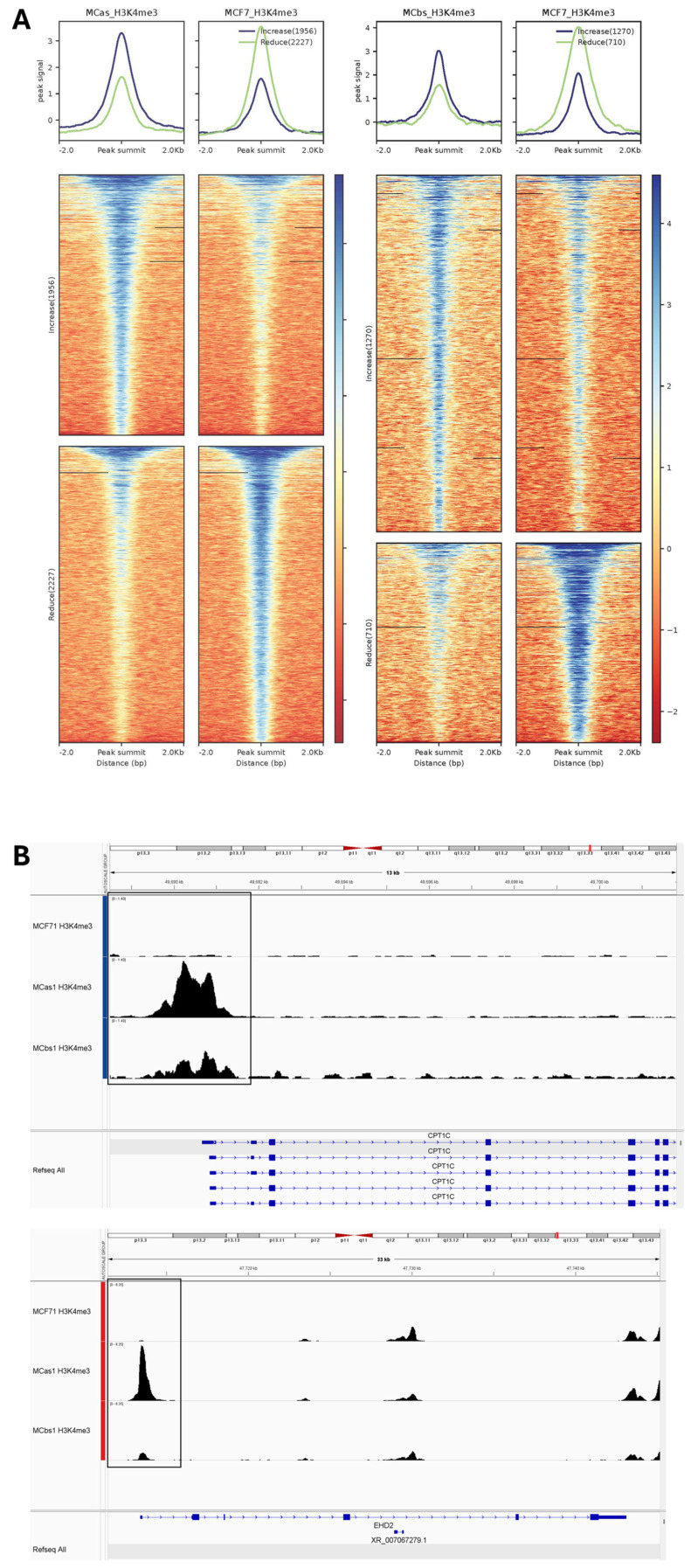
Chronic IL-1 exposure induces differential H3K4me3 binding in the MCF7 cell line. (**A**) Heat maps show peaks with differential H3K4me3 binding signals for MCas1 subline versus MCF71 parental (left) and MCbs1 subline versus MCF71 parental (right). Each peak region is displayed with 2Kb flanking regions around the summit of the peak. (**B**) Genome tracks show relative H3K4me3 enrichment at *CPT1C* and *EHD2* gene promoters (region framed in a black box) in MCF71 parental, MCas1, and MCbs1. Both genes are enriched for H3K4me3 promoter binding in the chronic IL-1 sublines relative to parental cells. Genome tracks were created using Integrative Genomics Viewer (v 2.12.3; IGV.org, UC San Diego, San Diego, CA, USA and Broad Institute Cambridge, MA, USA) with the reference human chromosome GRCh38/hg38. Arrows in the reference gene sequence (“Refseq All”) indicate transcriptional direction.

Figure 6A shows the global differential H3K4me3 binding in MCas1 or MCbs1 compared to MCF71 parental. Because the H3K4me3 histone modification is a key marker of active transcription, we overlayed H3K4me3 binding in the UTRs, promoter, and gene body with RNA sequencing. The overlay of shared DEGs in MCas1 and MCbs1 (881 basally high, 536 basally low) (Table S1, Tab B) with differential H3K4me3 binding in the UTRs, promoter, or gene body of annotated genes shows that 14 basally high genes, including *CPT1C* and *EHD2* (Figure 6B), are enriched for H3K4me3 binding (Table S2, Tab Q); 17 basally low genes show reduction in H3K4me3 binding (Table S2, Tab Q). Notably, only a small subset of DEGs in MCas1 and MCbs1 also showed differential H3K4me3 binding (Table S2, Tab Q), suggesting that IL-1 also regulates gene expression through other (epi)genetic or transcriptional mechanisms. In addition, differential H3K4me3 binding was found in genes that were not identified as DEGs in MCas1 or MCbs1, and vice versa (Table S2, Tab Q), likely due to the time lapse between RNA sequencing done at 28 weeks post-chronic IL-1 exposure and ChIP sequencing done at over 2 years post-chronic IL-1 exposure (see “Materials and Methods”), suggesting that chronic IL-1 genome regulation can be dynamic and transient. Together, these data suggest that altering promoter H3K4me3 histone levels is a mechanism by which chronic IL-1 exposure regulates gene expression.

## 3. Discussion

Chronic inflammation is a well-established driver of tumor progression, metastasis, and therapeutic resistance across many cancer types (e.g., IL-1, TNF-α, IL-6 pathways) [45]. In breast cancer, IL-1 signaling has been implicated in promoting tumor-promoting inflammation, angiogenesis, and metastasis, and targeting IL-1 receptor signaling has been proposed as a therapeutic strategy [46]. Still, much remains unknown regarding responses of hormone-receptor-positive breast cancer to prolonged IL-1. In this study, we investigated how chronic IL-1 exposure shapes the behavior of ERα+/PR+ breast cancer cells. Our findings show that while acute IL-1 signaling causes growth suppression, chronic IL-1 exposure selects for cell populations with attenuated inflammatory response while still remaining dependent on their hormone receptors and maintaining treatment sensitivity.

While the MCF7 and T47D parental cell lines induce the canonical IL-1 target gene, *LCN2*, upon acute IL-1 stimulation, this response is substantially reduced in the MCF7 and T47D chronic IL-1 sublines, indicating dampened inflammatory signaling (Figure 1 and Figure S1). However, chronic IL-1 exposure did not block acute IL-1-induced cytostasis or repression of ERα and PR (Figure 1 and Figure S1). Thus, chronic IL-1 exposure led to a partial insensitivity to acute IL-1 exposure rather than complete resistance (Figure 1 and Figure S1).

Importantly, despite exhibiting reduced sensitivity to IL-1, MCF7 and T47D chronic IL-1 sublines remained sensitive to other growth-limiting conditions like serum starvation (Figure 2 and Figure S2). Likewise, genetic silencing of ERα in MCF7 cells or PR in T47D cells significantly reduced proliferation and Ki67 expression in both parental and chronic IL-1 sublines (Figure 2 and Figure S2). This demonstrates that despite prolonged inflammatory stress and altered IL-1 responsiveness, these cells remain reliant on hormone receptor signaling for growth. This finding has important clinical implications, as it suggests that chronic IL-1 inflammation alone may be insufficient in driving hormone-independent growth in ERα+/PR+ BCa.

Consistent with preserved hormone dependence, MCF7 and T47D chronic IL-1 sublines retain sensitivity to standard BCa therapies. Fulvestrant treatment reduced cell accumulation, nuclear hormone receptor levels, and proliferation across all MCF7 and T47D parental and subline cell lines (Figure 2 and Figure S2). Similarly, docetaxel treatment effectively suppressed proliferation and cell viability in both MCF7 and T47D parental and chronic sublines (Figure 3 and Figure S3). This is in contrast to reports showing that acute IL-1 exposure (i.e., 24–96 h) leads to IL-1/γH2AX- or IL-1/ICAM1-dependent selection for docetaxel-resistant senescent or stem-like cells in lung [30] and head and neck [31] cancer models, respectively. And while acute IL-1 induces *ICAM1* expression in MCF7 and T47D cells (Table S1, Tab A and C), basally low *ICAM1* levels (Table S1, Tab D) and attenuated IL-1 sensitivity (Figure 1 and Figure S1) would be expected to subvert acute IL-1-dependent docetaxel resistance in the chronic IL-1 sublines. These results suggest that chronic IL-1 may not cause cells to evolve resistance to endocrine or chemotherapeutic agents, reinforcing the continued relevance of established therapies even in chronically inflamed tumors.

The MCF7 and T47D response to chronic IL-1 exposure was largely in contrast to what we found for the LNCaP prostate cancer (PCa) cell line, which showed strong attenuation of IL-1 sensitivity, reduced androgen receptor dependency, and anti-androgen resistance [16]. The MCF7 human BCa cell line was established from a pleural effusion, is classified as luminal A subtype, and represents noninvasive, early-stage, estrogen- and ERα-dependent BCa (REF). The T47D human BCa cell line was isolated from a pleural effusion, is classified as luminal A subtype, and expresses relatively high PR levels and mutated, nonfunctional p53 [47]. LNCaP cells were established from a lymph node metastasis and represent early-stage, androgen- and AR-dependent PCa disease [48]. Acute IL-1 treatment is significantly cytotoxic for LNCaP cells [19,23]. Thus, LNCaP cells that survive prolonged IL-1 exposure emerge more fit and able to adapt to androgen- and AR-independent growth conditions. While IL-1 is cytostatic for MCF7 cells, IL-1 is not cytotoxic for MCF7 or T47D cells (Figure 1), thus obviating the need to evolve survival strategies during chronic IL-1 exposure that could also support endocrine or chemotherapy resistance. This suggests that the extent of the response and the biological outcome following prolonged IL-1 exposure is dependent on the cell line genetic background. However, it should be noted that all cell lines, MCF7, T47D, LNCaP [16] C4-2 [49] and MDA-PCa-2b [19], evolved some level of attenuated IL-1 response following chronic exposure that may reflect a conserved regulatory mechanism among the different genetic backgrounds and an evolutionarily important cell adaption response.

At the transcriptional level, RNA-sequencing revealed substantial differences in acute and chronic IL-1 responsiveness and basal gene expression and differences among cell line backgrounds (Table S1). Despite these differences, a subset of IL-1-responsive and basal genes were shared between both cell line models, and gene ontology predicted that conserved inflammatory and cell cycle pathways are engaged. Thus, our novel subline models are powerful tools to investigate conserved mechanisms of acute and chronic IL-1 transcriptional reprogramming and will be valuable for identifying gene expression profiles that help define the state of the inflammatory TME in patient tumors.

Persistent transcriptional changes are frequently linked to epigenetic mechanisms, leading us to investigate histone modifications associated with active and repressive chromatin states in MCF7 cells. By integrating RNA-seq and ChIP-seq datasets, we identified a subset of genes displaying corresponding alterations in both gene expression and chromatin remodeling (Table S2), indicating that chromatin remodeling may underlie transcriptional changes induced by chronic IL-1 exposure. For instance, ChIP-seq analysis of *CPT1C* and *EHD2* demonstrated increased enrichment of the active histone mark H3K4me3 at their promoter regions in chronic IL-1 sublines compared to parental cells, aligning with their elevated expression levels (Figure 5 and Figure 6; Tables S1 and S2) This correlation between histone modification and transcription was not consistent across all genes, underscoring the complexity of epigenetic regulation and the contribution of additional mechanisms influencing gene expression. These results also suggest that chronic IL-1 exposure introduces transient, as well as stable, epigenetic and transcriptional changes. Thus, it will be necessary to monitor gene expression patterns, acquired genetic mutations, and epigenetics changes in parallel during and after chronic exposure to better define how chronic IL-1 reprograms gene expression.

Using our novel chronic IL-1 sublines, we sought to identify stable changes in molecular and cell biology phenotypes that evolve during chronic IL-1 exposure and persist once the inflammatory stimulus is removed. But our time course (Figure 1 and Figure 5) and sequencing (Tables S1 and S2) experiments collectively indicate that, in addition to inducing stable changes in molecular and cell response, chronic IL-1 effects can also be transient. Equally as important as stable changes, transient changes can also influence cancer progression and treatment resistance. For example, during chronic IL-1α exposure, PR levels are highly repressed in T47D cells (Figure 1B), which would be expected to render the cells endocrine-resistant during active chronic inflammation. But once the inflammatory stimulus is removed, PR levels are restored along with endocrine sensitivity (Figure 1, Figure 2 and Figures S1 and S2). This transient regulation, as well as the noted differential response to IL-1 isoforms, highlights the dynamics and complexity of IL-1 signaling. Thus, it will be informative and important to identify both stable and transient changes in cell state and behavior during and following chronic IL-1 exposure, as well as identify both the conserved and distinct effects of chronic IL-1α and IL-1β on cancer cell behavior. Together, these intriguing investigations could ultimately inform BCa treatment strategies, including if and when to augment standard therapies with IL-1 antagonists.

Collectively, our study refines our understanding of how ERα+/PR+ BCa tumor cells adapt within chronically inflamed microenvironments by demonstrating that prolonged inflammatory stress leads to stable, yet incomplete, attenuation of IL-1 responsiveness while preserving hormone receptor dependence and treatment sensitivity. These findings support the idea that chronic IL-1 exposure acts as a selective pressure that reshapes inflammatory signaling in BCa cells. These findings underscore the continued clinical relevance of hormone receptor-targeted and chemotherapeutic strategies in inflamed breast tumors. More broadly, this work supports the need to better understand the dynamics and role of acute versus chronic inflammatory signaling to improve outcomes in hormone-receptor-positive breast cancer.

## 4. Materials and Methods

### 4.1. Cell Culture

MCF7 (HTB-22) and T47D (HTB-133) cells were obtained from American Type Culture Collection (ATCC; Manassas, VA, USA) and were grown in a 37 °C, 5.0% (*v*/*v*) CO2 incubator. MCF7 cells were cultured in Dulbecco’s Modified Eagle’s Medium (DMEM; Gibco/Thermo Scientific; Grand Island, NY, USA; 1185-092) supplemented with 10% (*v*/*v*) fetal bovine serum (FBS) (Corning Inc., Corning, NY, USA; 35-011-CV), 0.4 mM L-glutamine (L-glut; Gibco/Invitrogen, Paisley, PA, USA; 25030-081), and 10 U/mL penicillin G sodium and 10 mg/mL streptomycin sulfate (pen-strep; Gibco/Invitrogen, Grand Island, NY, USA; 15140-122). T47D cells were maintained in Rossman-Park-Memorial-Institute (RPMI) 1640 (Gibco, 11875-119) medium supplemented with 0.01 mg/mL human recombinant insulin (Thermo Fisher Scientific, Fair Lawn, NJ, USA; 12585-014) 10% FBE, 0.4 mM of L-glutamine, 10 U/mL of penicillin G sodium, and 10 mg/mL of streptomycin sulfate.

### 4.2. Chronic IL-1 Subline Generation and Maintanence

To establish stable chronic IL-1-exposed sublines, MCF7 cells were cultured in DMEM/10% FB Essence (FBE) containing 0.5 ng/mL IL-1α (GoldBio, St. Louis, MO, USA; 1110-01A-10) or 0.5 ng/mL IL-1β (Gold Bio, St. Louis, MO, USA; 1110-01B-10) for 24 weeks and T47D cells were cultured in RPMI 1640/10% FBE containing 0.5 ng/mL IL-1α or IL-1β for 24 weeks (28 weeks for the T47Dbs1 subline due to slowed proliferation and expansion). Following chronic IL-1 exposure, cells were moved out of IL-1-containing medium and maintained in normal growth medium without IL-1 for phenotype characterization. IL-1 concentration and exposure time are based on previous chronic IL-1 sublines generated in human cell line backgrounds [16,49]. Briefly, because IL-1 can be cytotoxic and cytostatic, we optimized the concentration and chronic exposure time to ensure the induction of IL-1 signaling without inhibiting the establishment of viable, proliferative cells. For rigor and reproducibility, we use the same IL-1 concentration for both isoforms and for all cell line backgrounds. We generated two independent sets of each chronic IL-1 subline and we indicate the number of times the cells were passaged in culture during the chronic IL-1 exposure: MCF7 chronic IL-1α sublines (MCas1 (9 passages in IL-1α for 24 weeks), MCas2 (14 passages in IL-1α for 24 weeks)), MCF7 chronic IL-1β sublines (MCbs1 (11 passages in IL-1β for 24 weeks), MCbs2 (14 passages in IL-1β for 24 weeks)), T47D chronic IL-1α sublines (T47Das1 (8 passages for 24 weeks), T47Das2 (19 passages for 24 weeks)), and T47D chronic IL-1β sublines (T47Dbs1 (11 passages in IL-1β for 28 weeks), T47Dbs2 (20 passages for 24 weeks)). Parental cell lines (MCF71 (14 passages in vehicle for 24 weeks), MCF72 (14 passages in vehicle for 24 weeks), T47D1 (8 passages in vehicle for 24 weeks), T47D2 (13 passages in vehicle for 24 weeks)) were cultured in vehicle control (1× phosphate-buffered saline, PBS) (Corning, Manassas, VA, USA; 21-040-CM) alongside the sublines. Cells were passaged when cells reached confluency or sub-confluency in order to expand cells and collect cells for analysis. Cell line authentication for MCF7 and T47D parental and subline cells was performed by STR profiling by the DNA Genotyping Core, UTSW Medical Center. We hereby confirm that none of the cell lines used required any ethical approval for their use.

### 4.3. Cell Treatments

#### 4.3.1. Cytokines

Human recombinant IL-1α (GoldBio, St. Louis, MO, USA; 1110-01A-100) or IL-1β (GoldBio, St. Louis, MO, USA; 1110-01B-100) was resuspended in 0.1% bovine serum albumin (BSA) (Thermo Fisher Scientific, Fair Lawn, NJ, USA; BP 1600-1) in 1× phosphate-buffered saline (PBS; Corning, Manassas, VA, USA; 21-040-CM). Cells were treated with vehicle control (0.1% BSA in 1× PBS), IL-1α or IL-1β added to DMEM/10% FBE growth medium.

#### 4.3.2. Gene Silencing (siRNA)

Cells were treated with 70 nM *ERα* siRNA (Dharmacon, Lafayette, CO, USA; M-003401-04-0020) or 70 nM *PR* siRNA (Dharmacon, Lafayette, CO, USA; M-003433-01-0005). Cells were transfected using the TransIT-X2 Dynamic Delivery System (Mirius, Madison, WI, USA; MIR 6003) in 250 µL of DMEM/10% FBE growth medium. The next day, an additional 150 µL of DMEM/10% FBE was added to MCF7 cells or 150 µL of RPMI 1640/10% FBE was added to T47D cells. Cells were analyzed for phenotypes 3 days later for a total of 4 days in siRNA reagent.

#### 4.3.3. Serum Starvation

Cells were plated in DMEM/2.5% FBE (for MCF7) or RPMI/2.5% FBE (for T47D) growth medium and after 2–3 days, the medium was replaced with DMEM/0% FBE or RPMI 1640/0% FBE (serum starvation) or DMEM/10% FBE or RPMI 1640/10% (replete medium control).

#### 4.3.4. Fulvestrant (FULV)

Cells were plated and maintained in DMEM/10% FBE or RPMI 1640/10% FBE growth medium, and after 2–3 days, the medium was replaced with DMEM/10% FBE plus 100 nM fulvestrant (FULV; ApexBio, Houston, TX, USA; Cat. No. A1428) or vehicle control (ethanol).

#### 4.3.5. Docetaxel (DTX)

Cells were plated and maintained in DMEM/10% FBE or RPMI 1640/10% FBE growth medium, and after 2–3 days, the medium was replaced with DMEM/10% FBE plus 10 nM docetaxel (DTX; Millipore Sigma, St. Louis, MO, USA;148408-66-6,) or vehicle control (DMSO).

### 4.4. Protein Analysis

#### 4.4.1. Western Blot

Protein isolation and Western blot were performed as previously described [16]. Western blot densitometry was performed using ImageJ (v 1.53k, NIH). Protein bands were normalized to β-actin loading control.

#### 4.4.2. Antibodies

Primary antibodies: LCN2 (Cell Signaling Technology, Danvers, MA, USA; 44058S), ERα (Cell Signaling Technology, Danvers, MA, USA; 8644S), PR (Cell Signaling Technology, Danvers, MA, USA; 3153S), and β-actin (Santa Cruz, Santa Cruz, CA, USA; sc-69879). Secondary antibodies: sheep anti-mouse (Jackson ImmunoResearch Laboratories, Grove, PA, USA; 515-035-062), goat anti-rabbit (Abnova, Walnut, CA, USA; PAB10822).

#### 4.4.3. Immunofluorescence

Cells were fixed with paraformaldehyde (PFA) for 15 min, then permeabilized with 0.2% triton for another 15 min. Fixed cells were blocked with 2.5% BSA in 1× PBS at room temperature for at least 30 min. Antibodies were diluted in 2.5% BSA in 1× PBS. Cells were incubated in primary antibody overnight at 4 °C, washed with 1× PBS, and then incubated with fluorescently labeled secondary antibody overnight at 4 °C in the dark. Primary antibodies: ERα (Cell Signaling Technology, Danvers, MA, USA; 8644S), PR (Cell Signaling Technology, Danvers, MA, USA; 3153S), and Ki67 (Millipore, Darmstadt, Germany, MAB4190). Fluorescently labeled secondary antibodies: Alexa Fluor 568, goat anti-rabbit (Invitrogen, Waltham, MA, USA; A11011); Alexa Fluor 488, goat anti-mouse (Invitrogen, Waltham, MA, USA; A11001). Nuclei were stained with DAPI (Roche Diagnostics, Basel, Switzerland; 10,236,276,001). Immunostained cells were imaged at 10× magnification using a Nikon epifluorescence microscope (Nikon, Melville, NY, USA).

### 4.5. Cell Counts

Follow treatment, medium and cell debris were removed. Attached cells were fixed with 100% cold methanol for at least 15 min. The nuclei were then stained with DAPI (Roche Diagnostics, Basel, Switzerland; 10,236,276,001) and automated counting of nuclei was done using the Cytation3 Imaging Reader (BioTek, Winooski, VT, USA). Cell counts were normalized to day 0 for each cell line. 220,000 > *n* > 100 total cells counted per biological replicate. Error bars = ±STDEV of 3 biological replicates; *p*-value = * ≤0.05, ** ≤0.005, *** ≤0.0005.

### 4.6. DAPI Ratio

Following siRNA treatment, medium and cell debris were removed, and attached cells were fixed and (immuno)stained for analysis. To confirm siRNA efficacy in a cell population, the ratio of the number of ERα- or PR-positive nuclei to DAPI-stained nuclei was determined. To assess the effect on proliferation in a cell population, the ratio of the number of Ki67-positive nuclei to DAPI-stained nuclei was determined. Cells were counted from ten (10) 10× magnification microscopy fields for each of 3 biological replicates. Ratios were normalized to control siRNA. *n* > 400 total cells counted per biological replicate. Error bars = ±STDEV of 3 biological replicate; *p*-value = * ≤0.05, ** ≤0.005, *** ≤0.0005.

### 4.7. RNA Sequencing and Differential Gene Expression Analysis

#### 4.7.1. RNA Sequencing and Differential Gene Expression

Based on previous established sublines [16,19,49] to best capture stable, heritable changes in gene expression, the established MCF7 chronic IL-1 sublines (MCas1, MCbs1) along with parental (MCF71) were subsequently cultured in normal growth medium for 28 weeks before RNA sequencing, and the established T47D chronic IL-1 sublines along with parental were subsequently cultured in normal growth medium for 48 weeks (T47D1, T47Das1) and 16 weeks (T47Dbs1) before RNA sequencing. Listed are the number of times parental and sublines were passaged in normal growth medium before sequencing: MCF71, 21 passages; MCas1, 17 passages; MCbs1, 20 passages; T47D1, 24 passages; T47Das1, 29 passages; T47Dbs1, 14 passages. RNA-sequencing and differential gene expression analysis was done as previously described, with the following cut offs: log2 counts per million (CPM) ≥ 0, log2 fold change (FC) ≥ 0.6 or ≤−0.6, false discovery rate (FDR) ≤ 0.05, *p*-value ≤ 0.05 [50]. RNA-seq datasets generated for this study are available at GEO NCBI, accession GSE335261.

#### 4.7.2. Over-Representation Analysis

Over-representation analysis (ORA) of differentially expressed genes was performed using the WEB-based Gene SeT AnaLysis Toolkit (WebGestalt) site (http://www.webgestalt.org/; accessed on 16 May 2026). Gene lists were analyzed using the gene ontology biological process functional database. The parameters for the enrichment analysis are minimum/maximum genes in a category = 5/2000, FDR method = BH, number of categories to report = top 40. Other filters: method of interest: over-representation analysis; organism of interest: Homo sapiens; functional database: gene ontology, biological process: noRedundant; analyte type: gene/protein; reference set: genome; significance level: top 40.

#### 4.7.3. Gene Set Enrichment Analysis

Gene Sete Enrichment Analysis (GSEA) was performed using WebGestalt (http://www.webgestalt.org/; accessed on 16 June 2026 with all genes ranked from the differential expression analysis. Enrichment was assessed against the Homo sapiens gene ontology (GO) biological process and KEGG pathway databases using a minimum/maximum gene set size of 5/500 genes, 1000 permutations, and reporting of the top 15 enriched categories. An FDR ≤ 0.25 is considered significant [34].

### 4.8. RNA Analysis

#### 4.8.1. RNA Isolation and Reverse Transcription Quantitative PCR (RT-qPCR)

RNA isolation and RT-qPCR were performed as previously described [16].

#### 4.8.2. Primer Sequences

5′-3′: *Beta actin (β-actin)*, forward GATGAGATTGGCATGGCTTT, reverse CACCTTCACCGGTCCAGTTT; *Ras related dexamethasone induced 1 (RASD1)*, forward CACGCCTACCATCGAGGACT, reverse AACACCAGGATGAAAACGTCTC; *EH domain-containing protein 2 (EHD2)*, forward AGCTCAACGACCTGGTGAAG, reverse AGATGACGGGCAGTTTGAGG; *Keratin 13* (*KRT13*), forward TGAGATGGAGTGCCAGAACC, reverse CTGCTGAGGAAGGGAAACCAA; *Ceruloplasmin (CP)*, forward CCCTGGAACATACCAAACCCT, reverse CAGCCAGATTGGTGTCTTCATTT; *Zona pellucida-like domain containing 1 (ZPLD1)*, forward GCCCCTTCCTTATGCCGATT, reverse GGTTGGAGTCTCATCACTCCG; *Hydroxycarboxylic Acid Receptor 1 (HCAR1)*, forward GGCTGCGGACAGGTATTTCA, reverse CACGCAGAGATGGTTCTCCA; *Purkinje cell protein 4 (PCP4)*, forward TGATGAGCGACAAGGTGCT, reverse CACGTTCTGTCTCTGGTGCAT.

### 4.9. Chromatin Immunoprecipitation Sequencing

#### 4.9.1. Sequencing

MCF71, MCas1, and MCbs1 cell (sub)lines, which had been cultured in normal growth medium over the course of 2.5 years for 71, 71, and 73 passages, respectively, were analyzed for epigenetic modifications using chromatin immunoprecipitation (ChIP). ChIP assays were performed at the MD Anderson Cancer Center Epigenomics Profiling Core as described previously [51,52] with some modifications. Briefly, MCF71, MCas1, and MCbs1 cells were crosslinked in 1% formaldehyde for 10 min at room temperature, followed by incubation with 125 mM glycine for 5 min to quench formaldehyde. The cells were pelleted and washed twice with ice cold PBS. Chromatin was prepared by nuclear isolation followed by lysis. Lysates were sonicated using a Bioruptor^®^ Pico (Diagenode, Denville, NJ, USA) to obtain DNA fragments ranging from 200 to 600 bp and cleared by centrifugation at 16,000× *g* for 10 min at 4 °C. Supernatant (chromatin lysate) was incubated with either H3K4me1, H3K4me3 (both from Abcam, Waltham, MA, USA), H3K27ac (Active Motif, Carlsbad, CA, USA) or H3K27me3 (Diagenode, Denville, NJ, USA) antibodies conjugated with DynabeadsTM Protein A beads (Invitrogen) overnight at 4 °C. The next day, bead-conjugated antibody-bound chromatin was collected using DynaMagTM-2 Magnet (Invitrogen) and washed extensively, followed by reverse crosslinking and DNA purification. Input and ChIP DNA libraries were prepared using the NEBNext^®^ Ultra™ II DNA Library Prep Kit for Illumina^®^ (New England Biolabs, Ipswich, MA, USA) following the manufacturer’s instructions and subjected to next-generation sequencing to obtain 25 million 50 bp paired-end reads per sample. ChIP-seq datasets generated for this study are available at GEO NCBI, accession GSE337025.

#### 4.9.2. Bioinformatics

Raw ChIP-seq reads were first evaluated for quality using fastQC (v 0.12.1), which provides metrics including per-base sequence quality, GC content, duplication levels, read length distribution, k-mer profiles, and potential adapter contamination. Adapter sequences and low-quality bases were then trimmed using fastp (v 0.24.0) to produce high-quality clean reads. For fastp, the adapters of pair-end data can be trimmed automatically by per-read overlap analysis, which seeks the overlap of each pair of reads. The processed reads were aligned to the human reference genome (GRCh38) using bwa (v 0.7.19). Then, samtools (v 1.21) was used for sorting, indexing, and mapping quality assessment. Duplicates were marked using picard (v 3.4.0). Macs2 (v 2.2.6) was applied to identify peaks through peak calling. Differential binding site analysis was performed using MAnorm (v 3.16.0) [53] software, which allowed us to identify differential binding peaks. The threshold MA values (similar to log2 fold change) higher than 1.0 or lower than −1.0 were used to determine the differential binding peaks.

#### 4.9.3. Heat Map

Deeptools software (v3.5.6) [54] was used to plot the heatmap of ChIP-seq signals around each H3K4me3 peak with differential binding signals selected by comparing peaks between MCas/MCbs and MCF7cell lines by MAnorm. Each peak region was displayed with 2Kb flanking regions around the summit of the peak.

## Data Availability

Sequencing data generated for this report is publicly available at GEO NCBI Accession GSE335261 (RNA sequencing) and GEO NCBI Accession GSE337025 (ChIP sequencing).

## Supplementary Materials

The following supporting information can be downloaded at https://www.mdpi.com/article/10.3390/ijms27136039/s1.
