# Supplementary material for: Chronic IL-1 Exposure Attenuates IL-1 Response and Alters Gene Expression Regulation While Maintaining Therapeutic Sensitivity in BCa Cell Lines
Source: Int J Mol Sci. 2026 Jul 5;27(13):6039. doi: 10.3390/ijms27136039 (PMC13362516; doi:10.3390/ijms27136039)
Supplement: Supplementary file 1 [file ijms-27-06039-s001.zip › Falah et al_SupplementalFigures_REVISION_26Jun2026.pdf]

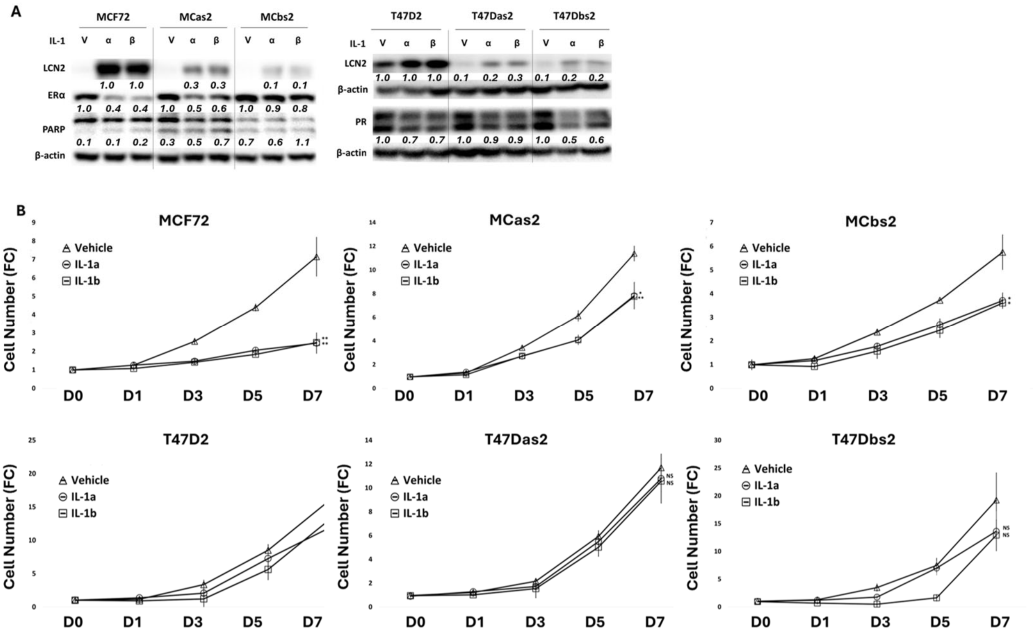

Figure S1. Chronic IL-1 selects for BCa cells that both lose and retain acute IL-1 responses. (A) MCF72 and T47D2 parental control cell lines, and their respective corresponding chronic IL-1 sublines, MCas2, MCbs2, T47Das2, and T47Dbs2, were treated acutely for 4 days with 25 ng/mL IL-1α or IL-1β. Protein was isolated and Western blot was performed for ERα and PR hormone receptors and the canonical IL-1-induced gene, *Lipocalin 2* (*LCN2*), as a surrogate for IL-1 sensitivity. In comparison to parental control cells, the MCF7 and T47D chronic IL-1 sublines show attenuated response to acute IL-1 repression of LCN2 but, with the exception of MCbs2, maintain sensitivity to acute IL-1 repression of ERα and PR. MCF7 parental and subline cells were also analyzed for PARP cleavage, an indicator of apoptosis. Acute IL-1 does not induce considerable PARP cleavage, suggesting the acute IL-1 is not cytotoxic for MCF7 parental or subline cells. (B) MCF72 and T47D2 parental and sublines cells were treated with vehicle control, 25 ng/mL IL-1α, or 25 ng/mL IL-1β for 7 days. Cells were fixed and stained with the DAPI nuclear marker. DAPI-stained cells were counted before IL-1 treatment (day 0, "D0") and on 1, 3, 5, and 7days after treatment, and the average fold change (FC) in cell counts was plotted. IL-1-treated MCF72, MCas2, and MCbs2 cells show reduced proliferation rate, while IL-1 had comparably little or no effect on T47D2 parental and subline cells. Cell counts were normalized to day 0 for each cell line. N = 3 biological replicates; error bars = +/−STDEV; p-value = * ≤0.05, ** ≤0.005, *** ≤0.0005. Western blot densitometry was performed using Image J and protein bands were normalized to β-actin loading control. LCN2 bands were further normalized to parental control for each cytokine, ERα and PR bands were further normalized to the vehicle control within the cell line or subline, and the ratio of cleaved/full length PARP was determined.

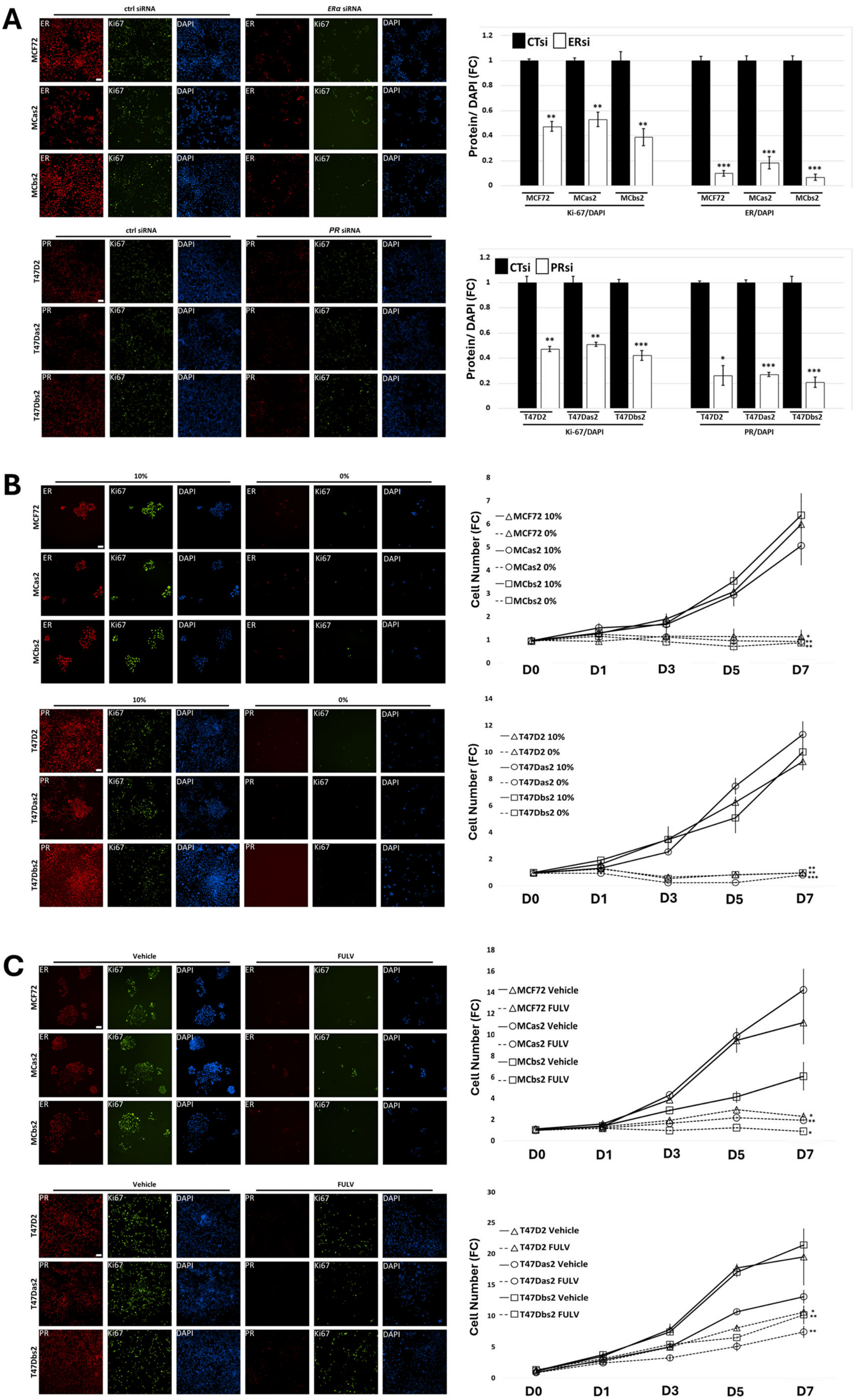
A
ctrl siRNA
ERα siRNA
MCF72
MCas2
MCbs2
CTsi
ERsi
Protein/ DAPI (FC)
Ki-67/DAPI
ER/DAPI
PR siRNA
T47D2
T47Das2
T47Dbs2
PRsi
PR/DAPI
B
10%
0%
Cell Number (FC)
MCF72 10%
MCF72 0%
MCas2 10%
MCas2 0%
MCbs2 10%
MCbs2 0%
D0
D1
D3
D5
D7
T47D2 10%
T47D2 0%
T47Das2 10%
T47Das2 0%
T47Dbs2 10%
T47Dbs2 0%
C
Vehicle
FULV
MCF72 Vehicle
MCF72 FULV
MCas2 Vehicle
MCas2 FULV
MCbs2 Vehicle
MCbs2 FULV
T47D2 Vehicle
T47D2 FULV
T47Das2 Vehicle
T47Das2 FULV
T47Dbs2 Vehicle
T47Dbs2 FULV

Figure S2. Chronic IL-1 sublines maintain dependency on ERα and PR hormone receptors. MCF72, T47D2, and subline cells were (A) transfected with 70nM control siRNA, *ERα* siRNA, or *PR* siRNA for 4 days; (B) grown in 10% or 0% serum for 0, 1, 3, 5, and 7 days; or (C) treated with 100 nM fulvestrant (FULV) for 0, 1, 3, 5, and 7 days. Treated cells were fixed, DAPI-stained, and co-immunostained for ERα or PR and the proliferation maker, Ki67. To determine the treatment effect on cell proliferation, the ratio cells positive for ERα/DAPI, PR/DAPI, or Ki67/DAPI were plotted (A), or the number of DAPI-stained cells counted over time (B, C). siRNA, serum starvation, and FULV treatment reduce ERα, PR, and Ki67 nuclear accumulation and proliferation in MCF72, T47D2, and subline cells. DAPI ratios were normalized to control siRNA and cell counts were normalized to day 0 for each cell line. N = 3 biological replicates; error bars = +/−STDEV; p-value = * ≤0.05, ** ≤0.005, *** ≤0.0005. Images: scale bar = 100 μm. D = day, FC = fold change.

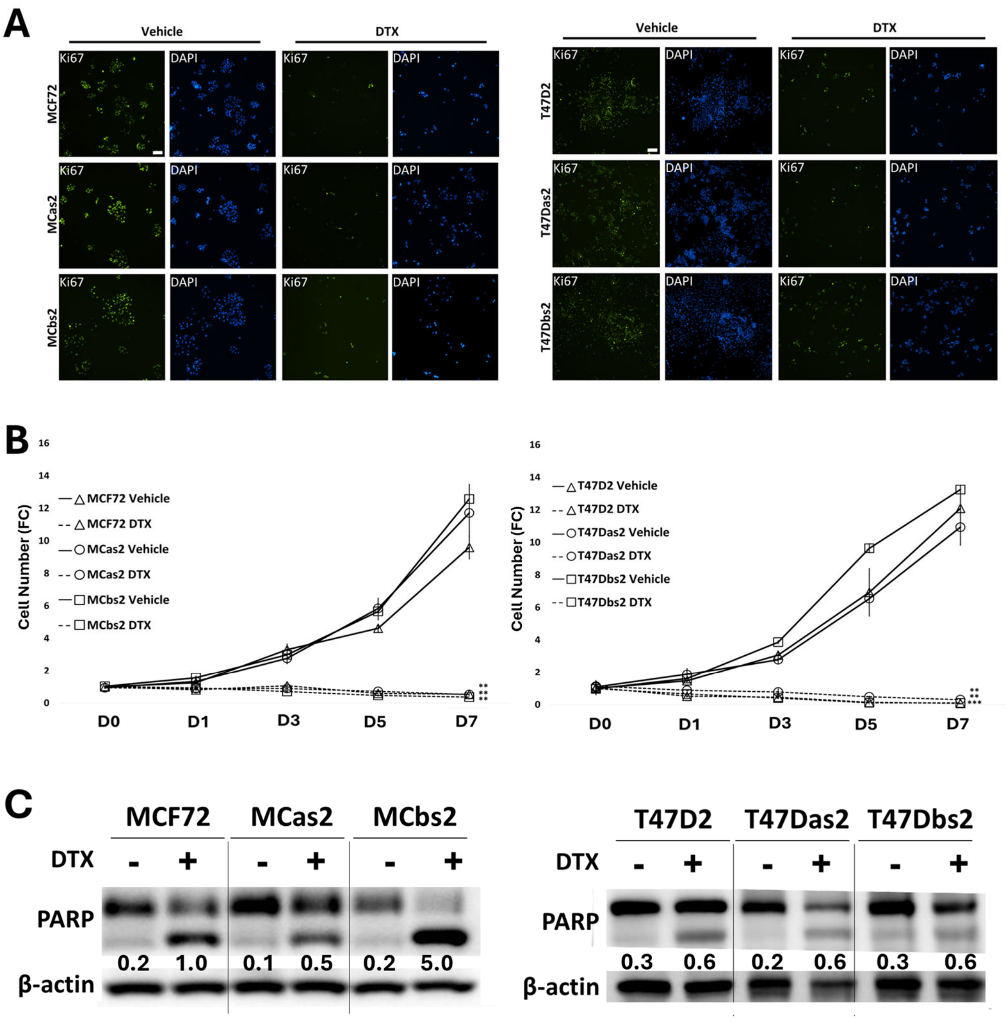

Figure S3. BCa chronic IL-1 sublines remain sensitive to chemotherapy. (A) MCF72, T47D2, and subline cells were treated with 10 nM docetaxel (DTX) for 3 days and co-stained with Ki67 and DAPI to determine the effect on proliferation. (B) MCF71, T47D1, and subline cells were treated with 10 nM DTX for 0, 1, 3, 5, and 7 days. Fixed, DAPI-stained cells were counted before treatment (day 0, "D0") and on 1, 3, 5, and 7 days after treatment, and the average fold change (FC) in cell counts was plotted. (C) MCF72, T47D2, and subline cells were treated for 3 days with 10 nM DTX and analyzed for PARP cleavage by Western blot to assay for apoptosis. DTX treatment reduces Ki67 staining and DAPI cell counts over time, and induces PARP cleavage in the MCF72, T47D2, and sublines. Cell counts were normalized to day 0 for each cell line. N = 3 biological replicates; error bars = +/−STDEV; p-value = * ≤0.05, ** ≤0.005, *** ≤0.0005. Images: scale bar = 100 μm. Western blot densitometry was performed using Image J and protein bands normalized to β-actin loading control and the ratio of cleaved/full length PARP was determined.
